# Individual hurricane evacuation intentions during the COVID-19 pandemic: insights for risk communication and emergency management policies

**DOI:** 10.1007/s11069-021-05064-2

**Published:** 2021-10-20

**Authors:** W. J. Wouter Botzen, Jantsje M. Mol, Peter J. Robinson, Juan Zhang, Jeffrey Czajkowski

**Affiliations:** 1 Institute for Environmental Studies, Vrije Universiteit, 1081 HV Amsterdam, The Netherlands; 2 Utrecht University School of Economics (U.S.E.), Utrecht University, Utrecht, The Netherlands; 3 Risk Management and Decision Processes Center, The Wharton School, University of Pennsylvania, Philadelphia, USA; 4 Center for Research in Experimental Economics and Political Decision Making (CREED), University of Amsterdam, Amsterdam, The Netherlands; 5 College of Business, Eastern Kentucky University, Richmond, USA; 6 Center for Insurance Policy and Research, National Association of Insurance Commissioners (NAIC), Kansas City, USA

**Keywords:** COVID-19, Evacuation, Hurricane preparedness, Pandemic, Risk perception

## Abstract

The U.S. 2020 hurricane season was extraordinary because of a record number of named storms coinciding with the COVID-19 pandemic. This study draws lessons on how individual hurricane preparedness is influenced by the additional risk stemming from a pandemic, which turns out to be a combination of perceptions of flood and pandemic risks that have opposite effects on preparedness behavior. We conducted a survey in early June 2020 of 600 respondents in flood-prone areas in Florida to obtain insights into households’ risk perceptions and preparedness for the upcoming hurricane season under COVID-19. The results show that concerns over COVID-19 dominated flood risk perceptions and negatively impacted people’s evacuation intentions. Whereas hotel costs were the main obstacle to evacuating during Hurricane Dorian in 2019 in the same geographic study area, the main evacuation obstacle identified in the 2020 hurricane season is COVID-19. Our statistical analyses investigating the factors influencing evacuation intentions show that older individuals are less likely to evacuate under a voluntary order, because they are more concerned about the consequences of becoming infected by COVID-19. We observe similar findings based on a real-time survey we conducted in Florida with another group of respondents under the threat of Hurricane Eta at the end of the hurricane season in November 2020. We discuss the implications of our findings for risk communication and emergency management policies that aim to improve hurricane preparedness when dealing with additional health risks such as a pandemic, a situation that may be exacerbated under the future climate.

**Supplementary Information:**

The online version contains supplementary material available at 10.1007/s11069-021-05064-2.

## Introduction

It has been projected that climate change may increase the risks of flooding due to sea-level rise and a possible increase in the severity of hurricanes (IPCC 2014). Therefore, adaptation policies such as purchasing insurance, taking risk reduction measures, and evacuating from a storm and flood threat should focus on improving individual preparedness for hurricanes to limit their destructive impacts. However, experience during the 2020 hurricane season shows that a pandemic may hamper hurricane preparedness, especially concerning evacuation. For instance, during the threat of Hurricanes Laura and Hanna in the United States, it was expected that those who did evacuate could cause a surge in COVID-19 cases with preparations for the pandemic leading to transport disruptions and difficulties in providing adequate shelter accommodation (Schulz et al. 2020). Moreover, many individuals may be less likely to evacuate during a storm threat when they are concerned about COVID-19 infections and their consequences, given that when people evacuate to hotels or shelters proper social distancing may not be possible. Previous studies have in fact shown that without the COVID-19 pandemic, natural disasters have already resulted in further spreading of infectious diseases attributed to the crowding of people, for example, in shelters (Ivers and Ryan 2006; Lemonick 2011; Shukla et al. 2018).

Insights into the influence of pandemics on hurricane preparedness can provide relevant information for risk communication and emergency management policies, because pandemics are likely to occur more often in our globalized economy (Philips et al. 2020). Moreover, climate change may exacerbate the risks of certain infectious diseases in addition to increasing the frequency and severity of extreme weather (IPCC 2014). Hence, lessons can be drawn for natural disaster risk management strategies from the 2020 hurricane season with record-breaking hurricane activity (NOAA 2020) that coincided with a pandemic. To our knowledge, this study is one of the first empirical analyses that draws lessons on how hurricane preparedness is influenced by a pandemic.

A similar recent study to ours is Collins et al. (2021), who conducted a survey of about 7,000 residents in Florida and found that these households were less likely to evacuate to a shelter in the 2020 hurricane season with COVID-19, compared with the pre-COVID-19 situation. Collins et al. (2021) further found that most of their respondents felt that being in a shelter during COVID-19 times posed a higher risk than enduring a hurricane in their home, highlighting the important role COVID-19 may play in individuals evacuation decisions. We conducted a survey around the same time in June 2020 in Florida to examine individuals’ general evacuation intentions at the start of the hurricane season, irrespective of whether this is to a shelter or some other place. In particular, we examine how evacuation intentions are independently influenced by flood risk perceptions and COVID-19 risk perceptions using regression and mediation analyses that we employ to further our understanding of how a person’s socio-demographic profile can influence evacuation intentions through these perceptions. This moves beyond the simple descriptive analyses by Collins et al. (2021). We also assess evacuation obstacles during the COVID-19 pandemic and how these compare to the 2019 hurricane season without COVID-19 that we collected using an earlier survey conducted in the same sample areas in February 2020. Moreover, we collected similar data at the end of the hurricane season using a real-time survey that was conducted when Hurricane Eta approached Florida in November 2020. These additional data allow us to examine if we find a similar influence of COVID-19 on evacuation intentions at the beginning and the end of the hurricane season.

In this paper, specific attention is paid to older people who may be more vulnerable to both low rates of evacuation and becoming very ill due to the coronavirus (Meng et al. 2020). A review by Huang et al. (2016) of actual evacuation studies found that 41% reported a significantly negative correlation between age and evacuation, while the other 59% reported a non-significant correlation. A number of factors can cause a negative relation between age and evacuation, e.g., lack of mobility, pre-existing health conditions, limited social networks, low income, and poor vision and hearing (Cohen and Mulvaney 2005; Rosenkoetter et al. 2007; Smith et al. 2009; Nakanishi et al. 2019; Dostal 2015). We study whether concerns about the consequences of becoming infected by COVID-19 are an additional barrier to evacuation for older people, who are also more likely to experience adverse health impacts from hurricanes (Jenkins et al. 2007). This focus is relevant since Collins et al. (2021) found that older people were more likely to believe that the threat of COVID-19 in shelters is more dangerous than the threat of a hurricane.

Based on the survey that we conducted in early June 2020 of 600 respondents residing within coastal regions of Florida, our results show that when it comes to the factors that influence evacuation intentions, flood risk perceptions are overshadowed by perceived risks related to COVID-19. Moreover, the main obstacle to evacuating during a storm threat changed from hotel costs in the 2019 hurricane season to COVID-19 in the 2020 hurricane season. Although these results were obtained from two different data collection methods and samples, they are indicative that COVID-19 became an important obstacle for evacuation during the pandemic. In particular, older people are less likely to evacuate due to deeper concerns about the consequences of becoming infected by COVID-19. We draw several implications from these findings for risk communication and emergency management policies that should be part of a broader adaptation strategy to limit impacts from future hurricanes. Our policy implications link to hurricane preparedness guidance with COVID-19 considerations that have been issued by various organizations and federal agencies, such as the American Red Cross (2020) and FEMA (2020), and state and local governments (NAIC/CIPR Research Library 2020).

## Data collected with surveys of coastal residents in Florida

**Fig. 1:**
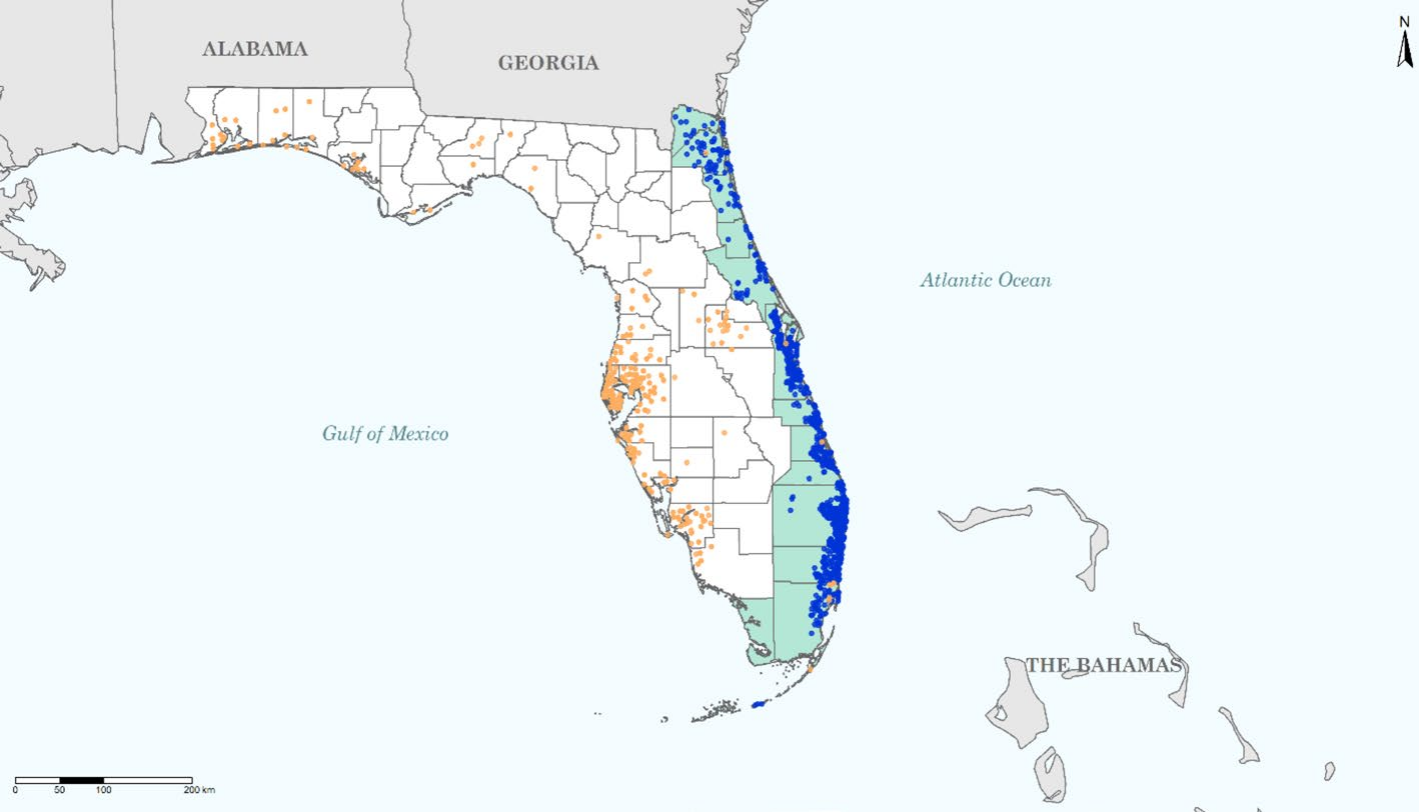
Locations of respondents in Florida to our surveys conducted in February 2020 (in blue dots), June 2020 (in green counties) and November 2020 (in orange dots)

This study is primarily based on a survey we conducted in early June 2020 of 600 respondents of coastal residents in Florida. The survey aimed to obtain insights into individual risk perceptions and hurricane preparedness for a hurricane season under a pandemic. The survey was conducted online using a representative sample of households living in the same areas as respondents to previous surveys we conducted in both August 2019 as well as February 2020 to analyze evacuation behavior related to Hurricane Dorian.^1^ Figure 1 shows the location of respondents in our June 2020 survey in blue dots. The sample was randomly drawn by a specialized survey company from an online consumer panel of residents in areas that were forecasted by the National Hurricane Center to be potentially hit by Hurricane Dorian in 2019. Although Hurricane Dorian eventually did not make landfall in Florida in 2019, it was a major threat when it approached the Florida coasts with winds speeds up to category 5. We sampled these areas for our June 2020 survey since they faced a substantial hurricane risk, and residents of these areas have likely recently considered evacuation. Moreover, this allows for a comparison of evacuation obstacles between a pre-pandemic hurricane season (February 2020 survey) and a post-pandemic hurricane survey (June 2020 survey) using households’ responses in the same areas.

The average age of respondents in our June 2020 survey is 48 years, with an average household income of $74,546 per year before taxes,^2^ and 66% are female. Respondents reported their highest level of education as follows: some high school (2%), high school graduate (17%), some college (26%), college graduate (36%), and post graduate (18%). Compared with the sample of the February 2020 survey, respondents to the June 2020 survey are 14 years younger on average, which may be explained by the data collection method. Older people are perhaps less likely to participate in online surveys than phone surveys. The February 2020 survey collected a significant portion of responses through phone questionnaires.

Moreover, we collected similar data on evacuation intentions and influencing factors at the end of the hurricane season using a real-time survey that was conducted when Hurricane Eta approached Florida in November 2020. Although Eta approached the U.S. as a hurricane, it was downgraded to a tropical storm when it made landfall in Florida on November 7 and again on November 12 and caused flooding in various areas. Total U.S. losses of Eta amounted to about $1.1bn (Aon 2020). This survey was completed by 844 respondents between 10 and 11 November before the second landfall of Eta. Figure 1 shows the location of our November 2020 survey respondents in orange dots. The sample was drawn by a specialized survey company and includes areas of the coast that could be impacted by wind and flooding caused by Eta. The average age of respondents in our November 2020 survey is 47 years, with an average household income of $40,134 per year before taxes,^3^ and 69% are female. Respondents reported their highest level of education as follows: some high school (3%), high school graduate (26%), some college (29%), college graduate (30%), and post graduate (12%). Compared with the June 2020 survey, our respondents to the November 2020 survey have a lower income and education level, but are similar in terms of age and gender.

Table 1 defines the variables used in our statistical analyses that were coded the same in the three surveys. To elicit these variables, respondents faced several questions in relation to: their risk perceptions associated with flooding and COVID-19, voluntary evacuation intentions, trust in the government response to COVID-19, as well as age, education, income, length of residence and gender. The overall selection of these questions allows us to identify to what extent perceptions of COVID-19 hamper evacuation, controlling for other potential determinants of evacuation, such as perceptions of the primary threat posed by a hurricane to households, i.e., flooding, and their socio-demographic profile. Our focus on risk perceptions is motivated by economic and psychological theories of individual decision-making under risk that point toward the important role of risk perceptions or threat appraisals in individual protective behaviors. Examples are Subjective Expected Utility Theory and Protection Motivation Theory (e.g., Botzen et al. 2015; Bubeck et al. 2012).

**Table 1:** Coding of variables used in our regression models

Variable	Coding
Worry about flooding	*I am worried about the danger of a flood at my current residence* 1 = strongly disagree to 5 = strongly agree
Perceived flood probability ^a^	*What is your best estimate of how often a flood will occur at your home?* categorical, 1 = less often than 1/1,000 years to 7 = more often than 1/10 years
Age	*How old are you?* in years
Education	*What is your highest completed level of education?* 1 = some high school to 5 = post graduate
Income	*Which of the following describes your total household income for 2019 before taxes?* 1 = less than $10,000 to 6 = $125,000 or more
Length of residence	*How long have you lived in your home (in years)?*
Gender	*Was the respondent male of female?* female = 1, male = 0
Voluntary evacuation intention	*Please tell me if you are extremely likely, likely, somewhat likely or not at all likely to evacuate to a safer place this hurricane season if a voluntary evacuation were to be ordered for your county* 1 = not at all likely to 4 = extremely likely
Perceived coronavirus infection probability	“*How likely do you think it is that you will personally be infected by the coronavirus?”* 1 = very unlikely to 5 = very likely
Concern about COVID-19	*The probability of being infected by the coronavirus is so low that I am not concerned about its consequences* 1 = strongly agree to 5 = strongly disagree (higher numbers indicate more concern)

^a^Our results are robust to this alternative coding of this variable on a 4 point scale: 1 = best estimate < 1 in 1,000 years, 2 = 1 in 100 years > best estimate ≥ 1 in 1000 years, 3 = 1 in 10 years > best estimate ≥ 1 in 100 years, 4 = best estimate ≥ 1 in 10 years

Flood risk perceptions were elicited according to both qualitative and quantitative measures. The quantitative question displayed probability answer options on a logarithmic scale, which has been shown to perform well in terms of eliciting low likelihood risks (Woloshin et al. 2000; de Bruin et al. 2011). Moreover, concern about the consequences of flooding and worry about the danger of a flood at respondents’ homes were asked using items adopted from some previous studies (Robinson and Botzen 2018, 2019; Botzen et al. 2015).

Concern and worry related to COVID-19 were elicited on similar formats as those related to flooding. A qualitative measure was also selected for the perceived COVID-19 infection probability, given the expected difficulty that respondents may have with attaching a numeric probability to the likelihood of becoming infected with the novel coronavirus.^4^ Furthermore, at the time we conducted our survey in early June, COVID-19 had mainly caused high infections in areas other than Florida, such as New York.^5^ Nevertheless, our respondents were highly concerned about the consequences of becoming infected by COVID-19 (see Sect. 3), which makes it relevant to examine how coronavirus risk perceptions influence evacuation intentions. The expectation of the level of illness respondents would experience upon falling ill from COVID-19 was asked on a qualitative response scale.

Our main outcome variable, voluntary evacuation intention, was asked on a qualitative ordinal response scale. Therefore, we treat this intention as an ordinal dependent variable in our statistical analyses, using ordered probit models. This method accounts for the ordinal nature of intentions, avoids having predicted probabilities that fall outside the unit interval (Cameron and Trivedi 2005), and makes no assumptions regarding the interval distances between answer options (Liddell and Kruschke 2018). As an aside, we do not dichotomize this ordinal variable as this would discard potentially useful data and reduce statistical power (Fitzsimons 2008). The exact way in which we derived the variables for the regression and mediation analyses in Table 1 can be found in the Supplementary Information.

## Results

### Individual perceptions of flood and COVID-19 risks at the start of the 2020 hurricane season

**Fig. 2:**
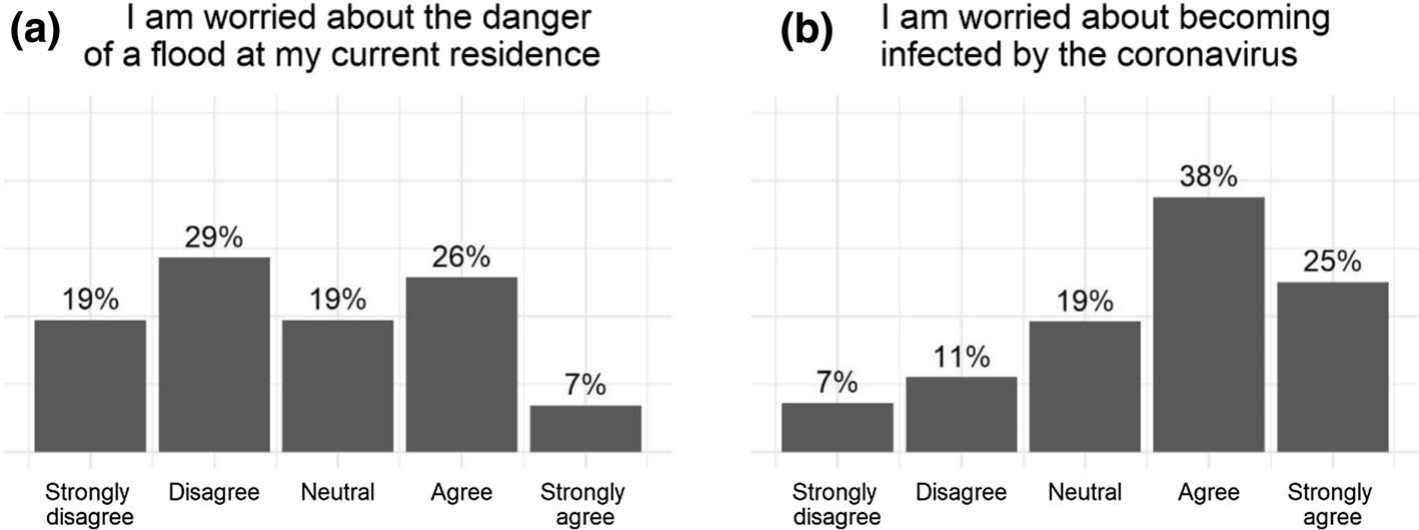
Responses to statements about worry of flooding (panel a) and COVID-19 (panel b) (based on the June 2020 survey)

A comparison of perceptions of COVID-19 and hurricane-related risks shows that perceptions of COVID-19 risks at the start of the 2020 hurricane season exceed those of flood risks. For example, more people are worried or strongly worried about COVID-19 risks (63%) than about flood risks (33%), as Fig. 2 illustrates. Moreover, only 21% disagree and 7% strongly disagree with the statement, “The probability of flooding is so low that I am not concerned about the consequences of a flood.” These percentages are 28% and 30%, respectively, for a similar statement about the consequences of being infected by the coronavirus. Individuals not only worry about the negative consequences of COVID-19, but also perceive high infection risks. For instance, 34% of respondents believe that it is likely or very likely they will become infected by COVID-19, and 39% expect to become very ill or extremely ill, once infected. More than half of the sample already experienced expenses because of COVID-19, mainly due to a loss of income. The vast majority of respondents (81%) are worried about the current economic situation.^6^ Previous research has shown that feelings toward risks are likely to affect how people prepare for a disaster. Examples are the worry about the consequences of a hazard or perceptions about whether or not the probability of experiencing a threat is high enough to trigger concern (Kunreuther and Pauly 2018; Botzen et al. 2019). In summary, our survey results indicate that the start of the 2020 hurricane season is dominated by concerns about COVID-19, which can influence hurricane preparedness activities.

### Evacuation intentions at the start of the 2020 hurricane season

When being asked about intentions to evacuate to a safer place under a voluntary evacuation order at the start of the hurricane season, 39% of respondents answer that it is likely or extremely likely they would evacuate (see Table 2).

**Table 2:** Please tell me if you are extremely likely, likely, somewhat likely or not at all likely to evacuate to a safer place this hurricane season if a voluntary evacuation were to be ordered for your county (based on the June 2020 survey)

	Evacuation intentions (%)
Not at all likely	37
Somewhat likely	25
Likely	24
Extremely likely	15

**Fig. 3:**
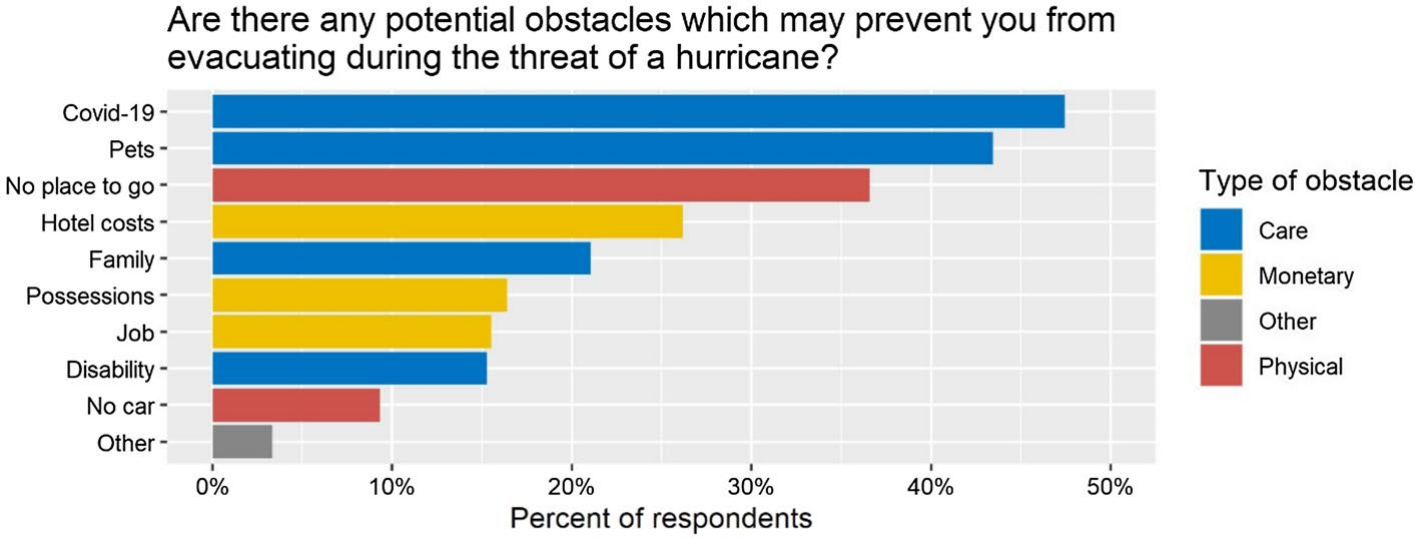
Percent of respondents who answered obstacles for evacuation, by obstacle (based on the June 2020 survey). Sample includes only respondents who reported at least one obstacle.

Descriptive statistics indicate that concern about COVID-19 is the most important obstacle for evacuation during the 2020 hurricane season. Both of our surveys in February and June 2020 contained a question about the obstacles for evacuation during a hurricane threat. More respondents indicated at least one potential obstacle in the June survey than in the February survey (75% versus 56%). The inability to pay for hotel costs was the most frequently mentioned obstacle during Hurricane Dorian (by 26% of the February 2020 survey respondents who had an obstacle). However, as Fig. 3 illustrates, hotel costs dropped to the number four obstacle during the 2020 hurricane season, although the percentage of respondents who list hotel costs as an obstacle remains stable at 26%. Instead, COVID-19 was mentioned the most frequently, by almost half of the respondents to the June survey who expected to experience any obstacles. Although these results were obtained from two different data collection methods and samples, they are indicative that COVID-19 became an important obstacle for evacuation during the pandemic.

We conduct a series of statistical analyses to examine how evacuation intentions under a voluntary order depend on socio-demographic characteristics and perceptions of the hurricane and COVID-19 risks. An ordered probit model of the intentions to evacuate voluntarily with only socio-demographic characteristics as explanatory variables finds that older people are significantly less likely to evacuate (Table 3).^7^ As a next step, we add perceptions of the hurricane and COVID-19 risks and length of residence as explanatory variables to the model to examine whether the significant relationship between age and intentions to evacuate still holds once these explanatory variables are controlled for. We include the variables: length of residence, perceived flood probability, worry about flooding, perceived coronavirus infection probability and concern about COVID-19 in model 2, since these variables are all significantly correlated with age (Pearson correlation coefficient *p* values < 0.05), and are potential predictors of voluntary evacuation intentions. Therefore, these variables may explain some of the relationship between age and voluntary evacuation intentions. Variables that are uncorrelated with age are omitted from the regression in model 2. In a mediation analysis (Table 4), we include only significant mediating variables when calculating indirect effects, which is the share of the relationship between age and voluntary evacuation that can be attributed to mediating variables. The other potential mediators are controlled for in a model that includes control variables.

**Table 3:** Ordered probit model of variables of influence on voluntary evacuation intentions (based on the June 2020 survey)

	Coefficients model 1	Coefficients model 2
*Socio-demographics*		
Age	− 0.010***	− 0.003
	(0.003)	(0.004)
Gender (1 = female)	− 0.247**	− 0.196
	(0.102)	(0.123)
Education	0.093*	0.118*
	(0.053)	(0.068)
Income	− 0.008	− 0.004
	(0.038)	(0.048)
Length of residence		− 0.016**
		(0.007)
*Flood risk perceptions*		
Perceived flood probability		0.008
		(0.039)
Worry about flooding		0.259***
		(0.062)
*COVID-19 perceptions*		
Perceived coronavirus infection probability		0.092*
		(0.050)
Concern about COVID-19		− 0.143***
		(0.047)
Observations	519	362
Log likelihood	− 682.4	− 443.6
Pseudo R^2^	0.017	0.081

***p < 0.01; **p < 0.05; *p < 0.1

Standard errors are shown in between parentheses below the coefficients. An ordered probit model is used to account for the ordinal nature of the dependent variable (1 = not at all likely to 4 = extremely likely to evacuate)

**Table 4:** Decomposition of the total effect of age on voluntary evacuation into direct and indirect effects via concern about COVID-19, worry about flooding and length of residence using the ordered probit model (based on the June 2020 survey)

	Without control variables	Including control variables
Total effect	− 0.012*** (0.003)	− 0.010*** (0.004)
Direct effect	− 0.005 (0.003)	− 0.003 (0.004)
Indirect effect	− 0.007*** (0.001)	− 0.007*** (0.002)
via concern about COVID-19	− 0.001** (0.001)	− 0.003** (0.001)
via worry about flooding	− 0.004*** (0.001)	− 0.003*** (0.001)
via length of residence	− 0.001* (0.001)	− 0.001 (0.001)
Mediation percentage	59.57	72.51
via concern about COVID-19	12.73	27.96
via worry about flooding	35.88	33.35
via length of residence	10.95	11.20
Observations	527	362

***p < 0.01; **p < 0.05; *p < 0.1

Coefficient estimates are provided with standard errors in parentheses on the same row

Control variables are: gender, education, income, perceived flood probability and perceived coronavirus infection probability

We find that the likelihood of voluntary evacuation significantly increases with worry about flooding, but significantly declines with concern about the consequences of becoming infected by COVID-19 and the length of residence (Table 3).^8^ Moreover, the independent effect of age on intentions to evacuate voluntarily becomes insignificant, indicating that the significant negative effect of age in the first model is an indirect effect, perhaps driven by perceptions of flood and COVID-19 risks as well as the length of residence. This result is examined in more detail using a mediation model (Table 4).^9^

Table 4 displays the total effect of age on voluntary evacuation, divided into a direct and indirect effect via concern about the consequences of becoming infected by COVID-19, worry about flooding, and length of residence. Overall, the total effect shows that older individuals have lower evacuation intentions. Controlling for concern about the consequences of becoming infected by COVID-19, worry about flooding, and length of residence leaves an insignificant direct effect of age. The indirect effect, which is the share of the relationship between age and voluntary evacuation that can be attributed to perceptions of COVID-19 and flood risks and length of residence, is explained by the coefficient estimate − 0.007 (*p* value < 0.01). Between 60 and 73% (depending on included control variables) of the relationship between age and voluntary evacuation is explained by concern about the consequences of becoming infected by COVID-19, worry about flooding, and length of residence.^10^ The two risk perception variables are statistically significant and explain a larger proportion of the relationship than the length of residence.

### Evacuation intentions during Hurricane Eta

When respondents to our real-time survey during the threat of Hurricane Eta were asked when they were going to evacuate to a safer place, 35% answered this is very unlikely, 27% answered unlikely, 10% answered likely, and only 6% answered very likely. We repeated the same analyses of evacuation intentions at the start of the 2020 hurricane season (that are reported in Tables 3 and 4) for evacuation intentions during Hurricane Eta, which hit Florida at the end of the hurricane season in November 2020. These results for evacuation during Hurricane Eta are reported in Tables 5 and 6. The ordered probit model results in Table 5 confirm our previous findings that evacuation intentions are negatively related to age (model 1), of which the significance declines to marginally significant in model 2 when risk perceptions are added. These findings again show that evacuation intentions are negatively related to concern about the consequences of becoming infected by COVID-19, and positively related to flood risk perceptions. Furthermore, although the sign of the coefficient estimate on the length of residence is the same in Table 5 as Table 3, this estimate is not significant in Table 5. Whereas, the perceived coronavirus infection probability is significantly positively related to evacuation intentions, which may be due to people with higher intentions to evacuate perceiving that they are more likely to become infected by COVID-19 in the event that an evacuation is in fact ordered.^11^

**Table 5:** Ordered probit model of variables of influence on voluntary evacuation intentions during Hurricane Eta (based on the November 2020 survey)

	Coefficients model 1	Coefficients model 2
*Socio-demographics*		
Age	− 0.017***	− 0.006*
	(0.00)	(0.00)
Gender (1 = female)	0.003	0.038
	(0.10)	(0.12)
Education	− 0.018	− 0.075
	(0.05)	(0.06)
Income	− 0.053	− 0.021
	(0.04)	(0.04)
Length of residence		− 0.001
		(0.01)
*Flood risk perceptions*		
Perceived flood probability		0.071*
		(0.04)
Worry about flooding		0.324***
		(0.05)
*COVID-19 perceptions*		
Perceived coronavirus infection probability		0.195***
		(0.05)
Concern about COVID-19		− 0.187***
		(0.05)
Observations	603	455
Log likelihood	− 689.4	− 464.2
Pseudo R^2^	0.032	0.131

***p < 0.01; **p < 0.05; *p < 0.1.

Standard errors are shown in between parentheses below the coefficients. An ordered probit model is used to account for the ordinal nature of the dependent variable (1 = not at all likely to 4 = extremely likely to evacuate)

**Table 6:** Decomposition of the total effect of age on voluntary evacuation during Hurricane Eta into direct and indirect effects via concern about COVID-19, worry about flooding and length of residence using the ordered probit model (based on the November 2020 survey)

	Without control variables	Including control variables
Total effect	− 0.017*** (0.003)	− 0.011*** (0.003)
Direct effect	− 0.010*** (0.003)	− 0.006* (0.004)
Indirect effect	− 0.007*** (0.002)	− 0.005*** (0.002)
via concern about COVID-19	− 0.001** (0.001)	− 0.003*** (0.001)
via worry about flooding	− 0.006*** (0.001)	− 0.002 (0.001)
via length of residence	− 0.000 (0.001)	− 0.000 (0.001)
Mediation percentage	42.36	42.04
via concern about COVID-19	7.11	26.82
via worry about flooding	32.56	14.34
via length of residence	2.68	0.89
Observations	600	455

***p < 0.01; **p < 0.05; *p < 0.1

Coefficient estimates are provided with standard errors in parentheses on the same row

Control variables are: gender, education, income, perceived flood probability and perceived coronavirus infection probability

Moreover, the mediation analysis results in Table 6 confirm that the main pattern of findings that are reported in Table 4 for evacuation intentions at the start of the 2020 hurricane season also hold for evacuation intentions among a separate sample who faced Hurricane Eta at the end of the 2020 hurricane season. More specifically, a large proportion of the relationship between age and voluntary evacuation intentions (in this case 42%) is explained by concern about the consequences of becoming infected by COVID-19 and worry about flooding. However, worry about flooding is only a statistically significant mediator without other control variables added to the model. Length of residence is also an insignificant mediator in Table 6 which is expected given the lack of significance of this variable in Tables 4 and 5.

## Policy implications and conclusion

The 2020 storm season, in which a record-breaking active hurricane season coincided with a pandemic, may be viewed as a learning experiment for risk communication and emergency management strategies that aim to limit hurricane impacts when more severe hurricanes in the future occur simultaneously with other health emergencies, such as a pandemic. Indeed, the results of our surveys of coastal residents in Florida conducted at the start and the end of the 2020 hurricane season show that hurricane preparedness is affected by the pandemic. The start of the 2020 hurricane season was dominated by concerns over COVID-19, which is an obstacle for evacuation. Moreover, older people, who are more concerned about the consequences of becoming infected by COVID-19, state lower evacuation intentions. This is apparent from evacuation intentions elicited among a sample at the start of the 2020 hurricane season, and confirmed by a real-time survey we conducted among another independent sample of respondents residing in the same state at the end of the hurricane season during the threat of Hurricane Eta. This should be taken into account by policies aimed at improving hurricane preparedness during a pandemic with a disease for which older people are more vulnerable. The majority of previous studies on evacuation that were not conducted during a pandemic did not observe a significant influence of age (Baker 1991; Sorensen 2000; Burnside et al. 2007; Meyer et al. 2018). Findings of a negative age effect by some (Huang et al. 2016) have been attributed to low mobility, poor health, limited social networks, and low income, as well as the length of residence (Morss et al. 2016). Our survey results show that evacuation behavior is different when a hurricane season coincides with a pandemic, because we observe a negative effect of age on evacuation intentions that is mainly caused by concerns over COVID-19 and worry about flooding.

Our results are consistent with an emerging literature that shows how COVID-19 jeopardizes the response to natural hazards (Cardil and de-Miguel 2020; Quigley et al. 2020; Ashraf 2021). Experiences of disasters during the pandemic illustrate that concerns about becoming infected by COVID-19, which prevent people from evacuating, may be justified. Hariri-Ardebili and Lall (2021) analyze various examples of flood disasters increasing COVID-19 infections around the world, including in Asia, Africa, and Western countries. Moreover, they discuss how COVID-19 obstructed emergency management responses, such as rescue operations (Hariri-Ardebili and Lall 2021). Although the impacts of the severe flood events in the summer of 2021 in Europe are still being studied, the first evidence suggests that the floods caused moderate increases in COVID-19 infections in flooded parts of the Netherlands (ENW 2021). Focused on the same geographical area as our study, Pei et al. (2020) show with model simulations that an increase in COVID-19 infections as a result of evacuations would be expected if a severe hurricane would strike Florida. Our findings of the impacts of COVID-19 risk perceptions on evacuation intentions in Florida are in line with results by Collins et al. (2021) that older people were more likely to believe that the threat of COVID-19 in shelters is more dangerous than the threat of a hurricane. Moreover, Borowski et al. (2021) show that individuals in the US are less willing to share rides to evacuate during a flood emergency if they have high concerns about becoming infected by COVID-19. Alam and Chakraborty (2021) examine evacuation decisions of households outside the US, namely in Bangladesh during cyclone Amphan, and also observe that individuals with high perceptions of COVID-19 risks are less likely to evacuate. Taken together, these findings highlight the need to account for health concerns in natural disaster risk management policies during a pandemic situation.

Adequate risk communication could be an important component of adaptation strategies to improve individual hurricane preparedness. For instance, our analyses of hurricane preparedness activities during Hurricane Dorian showed that risk awareness was an important driver of these activities (Botzen et al. 2020). Our survey at the start of the 2020 hurricane season reveals that risk communication by state governments, insurers, and insurance regulators reached a large number of respondents.^12^ Given the large influence of COVID-19 on evacuation intentions during the 2020 hurricane season, it is critical to refocus risk communication activities in times when the hurricane season coincides with a pandemic toward ensuring that people can safely evacuate by minimizing health risks. Examples during the COVID-19 pandemic are: including COVID-19 mitigation measures in hurricane preparedness kits, such as hand sanitizer and mouth masks, abiding by social distancing rules during an evacuation, and planning ahead to identify safe evacuation locations.

Moreover, governments and agencies can send more tailored communication messages to older people to alleviate their concerns over COVID-19 or improve their flood risk perceptions. Emergency management policies should create safe evacuation shelters where COVID-19 risks are well controlled and communicate their COVID-19 measures to the public to increase people’s confidence in shelters’ safety. Finally, our results show that the experience of living in a hurricane-prone area as proxied by the length of residence reduces evacuation intentions at the start of the hurricane season, which may be due to the experience of false alarms and near misses, such as Hurricane Dorian. Communication policies should stress that each storm is different, and the possibility of a direct hit by the next one should be taken seriously.

## Supplementary Information

Below is the link to the electronic supplementary material.

Supplementary file1 (DOCX 20 kb)

## Data Availability

The data used in this research are confidential.

## Footnotes

1 See Botzen et al. (2020) for the results of the August 2019 and February 2020 surveys (available on request). The February 2020 survey was completed by 255 respondents using a combination of phone and online questionnaires.

2 We converted the income categories to a continuous variable using the midpoint method. For example, the category $100,000 to $149,999 was recoded as 125,000. The number reported here is the average of this continuous variable.

3 See footnote 2.

4 This issue is less likely for the probability of flooding, given that respondents may be aware of this probability according to FEMA flood zone maps (https://msc.fema.gov/portal/home).

5 https://covid.cdc.gov/covid-data-tracker/#trends_dailytrendscases.

6 This variable is not further used in the analysis since worry about the economic situation is unlikely to influence evacuation intentions, but it does highlight that individuals are highly concerned about the impacts of COVID-19, which also encompasses its economic consequences.

7 The main results in Table 3 and Tables 4, 5 and 6 shown below are robust to including age as a dummy variable that represents people of age 65 and higher, which is the age group that is likely to experience more severe health consequences from infection by COVID-19.

8 We believe that concern about COVID-19 consequences is a stronger mediator of the relationship between age and voluntary evacuation intentions than the more general variable that reflects general worry about becoming infected with COVID-19, because we expect that older people are less likely to evacuate due to their concerns about the consequences of becoming infected by COVID-19, given that these tend to be more severe for aged individuals.

9 We also find that older individuals are less likely to state that they intend to evacuate if there is a mandatory evacuation order, but this is not due to perceptions related to COVID-19 and flooding. However, this may be due to other issues like poor mobility and health, or limited social networks, which we do not capture in the survey.

10 Correlation analyses show that a higher age is associated with higher concern about COVID-19 and length of residence, but a lower worry about flooding.

11 We estimated the models again without the perceived coronavirus infection probability to mitigate problems stemming from reverse causality. The conclusions remain the same.

12 Approximately 50% consulted information from these agencies to prepare for the hurricane season.
