# Supplementary material for: Individual hurricane evacuation intentions during the COVID-19 pandemic: insights for risk communication and emergency management policies
Source: Nat Hazards (Dordr). 2021 Oct 20;111(1):507–22. doi: 10.1007/s11069-021-05064-2 (PMC8526995; doi:10.1007/s11069-021-05064-2)
Supplement: Supplementary file 1 — Supplementary file1 (DOCX 20 kb) [file 11069_2021_5064_MOESM1_ESM.docx]

**Supplementary Information**

*Worry about flooding*

Please tell me if you strongly agree, agree, neither agree nor disagree, disagree or strongly disagree with the following: “I am worried about the danger of a flood at my current residence.”

 Strongly agree

 Agree

 Neither agree nor disagree

 Disagree

 Strongly disagree

 Not sure

*Perceived flood probability*

What is your best estimate of how often a flood will occur at your home?

 More often than 1 in 10 years

 Exactly 1 in 10 years

 Between 1 in 10 years and 1 in 100 years

 Exactly 1 in 100 years

 Between 1 in 100 years and 1 in 1000 years

 Exactly 1 in 1000 years

 Less often than 1 in 1000 years

 Not sure

*Voluntary evacuation intention*

Please tell me if you are extremely likely, likely, somewhat likely or not at all likely to evacuate to a safer place this hurricane season if a voluntary evacuation were to be ordered for your county.

 Extremely likely

 Likely

 Somewhat likely

 Not at all likely

 Not sure

*Perceived coronavirus infection probability*

How likely do you think it is that you will personally be infected by the coronavirus?

 Very likely

 Likely

 Not likely/not unlikely

 Unlikely

 Very unlikely

 Not sure

*Concern about COVID-19*

Please tell me if you strongly agree, agree, neither agree nor disagree, disagree or strongly disagree with the following: “The probability of being infected by the coronavirus is so low that I am not concerned about its consequences.”

 Strongly agree

 Agree

 Neither agree nor disagree

 Disagree

 Strongly disagree

 Not sure

*Age*

How old are you?

Age _____

*Education*

What is your highest completed level of education?

 Some high school

 High school graduate

 Some college

 College graduate

 Post graduate

 Refused

*Income*

Which of the following ranges best describes your total household income for 2019 before taxes?

 Less than $10,000

 $10,000 to $24,999

 $25,000 to $44,999

 $45,000 to $ 74,999

 $75,000 to $124,999

 $125,000 or more

 Don’t know

 Refused

*Length of residence*

How long have you lived in your home (in years)?

_____

*Gender*

Are you male or female?

 Male

 Female

 Refused
